# Imaging the Functional Neuroanatomy of Parkinson’s Disease: Clinical Applications and Future Directions

**DOI:** 10.3390/ijerph18052356

**Published:** 2021-02-28

**Authors:** Fulvio Lauretani, Yari Longobucco, Giulia Ravazzoni, Elena Gallini, Marco Salvi, Marcello Maggio

**Affiliations:** 1 Department of Medicine and Surgery, University of Parma, 43100 Parma, Italy; yari.longobucco@unipr.it (Y.L.); giulia.ravazzoni@unipr.it (G.R.); elena.gallini@unipr.it (E.G.); marco.salvi@unipr.it (M.S.); marcellogiuseppe.maggio@unipr.it (M.M.); 2 Cognitive and Motor Center, Medicine and Geriatric-Rehabilitation Department of Parma, University-Hospital of Parma, 43126 Parma, Italy

**Keywords:** Parkinson’s disease, neuroimaging, dopaminergic pathways, older persons

## Abstract

The neurobiology of Parkinson’s disease and its progression has been investigated during the last few decades. Braak et al. proposed neuropathological stages of this disease based on the recognizable topographical extent of Lewy body lesions. This pathological process involves specific brain areas with an ascending course from the brain stem to the cortex. Post-mortem studies are of importance to better understand not only the progression of motor symptoms, but also the involvement of other domains, including cognition and behavior. The correlation between the neuropathological expansion of the disease and the clinical phases remains demanding. Neuroimaging, including magnetic resonance imaging (MRI), positron emission tomography (PET), and single photon emission computed tomography (SPECT), could help to bridge this existing gap by providing in vivo evidence of the extension of the disorders. In the last decade, we observed an overabundance of reports regarding the sensitivity of neuroimaging techniques. All these studies were aimed at improving the accuracy of Parkinson’s disease (PD) diagnosis and discriminating it from other causes of parkinsonism. In this review, we look at the recent literature concerning PD and address the new frontier of diagnostic accuracy in terms of identification of early stages of the disease and conventional neuroimaging techniques that, in vivo, are capable of photographing the basal ganglia network and its cerebral connections.

## 1. Introduction

Significant progress has been made in understanding the neurobiology and progression of Parkinson’s disease (PD). Braak et al. [1] proposed neuropathological stages of this disease based on the recognizable topographical extent of Lewy body lesions. The pathological process involves specific brain areas starting from the brain stem to the cortex.

These post-mortem studies were an important step to better understand not only the progression of motor symptoms, but also the involvement of other clinical spheres, including cognition and behavior [2]. The correlation between the neuropathological extension of the disease and the clinical phases remains demanding.

Neuroimaging could help to bridge the gap by providing in vivo evidence of the extension of these disorders. These exams include the magnetic resonance imaging (MRI) [3], positron emission tomography (PET) [4], and the single photon emission computed tomography (SPECT) imaging techniques [5]. In the last decade, we observed an overabundance of reports regarding the sensitivity of neuroimaging techniques, even for capturing PD with MCI (mild cognitive impairment) [6]. All these studies were aimed at improving the accuracy of PD diagnosis and discriminating it from other causes of parkinsonism, as well as obtaining a surrogate marker of disease progression.

## 2. Materials and Methods

In this narrative literature review, we look at the historic keystone and main recent literature regarding the diagnostic conventional neuroimaging used to accurately assess PD and parkinsonism, especially at early stages, and to open new windows on frontiers of neuroimaging capable in vivo to detect the basal ganglia network and its cerebral connections. These new techniques are promising since they are more oriented toward early diagnostic-therapeutic approaches.

### 2.1. Structural Magnetic Resonance Imaging (sMRI)

The structural magnetic resonance imaging remained the key technique for differentiating neurodegenerative and symptomatic parkinsonism due to viral encephalitis, brain cancers, normal pressure hydrocephalus, and vascular diseases [3,4].

It could be possible to improve the accuracy of the MRI, passing from 1.5-T to 3-T and ultimately 7-T, in order to discriminate subjects with PD from healthy ones. The diagnostic approach requires the detection of iron concentration, which is increased during PD, as well as the shape and structure of the substantia nigra [7,8].

The increased iron content of the substantia nigra (SN) generally stands for the dopaminergic neuronal loss and MRI, which is able to identify the nigro-striatal signature of PD [9]. Abnormal iron deposition can also be found in atypical parkinsonism [10]. Recently, Kwon et al. also showed in a few patients with PD, specific structural alteration of the substantia nigra, represented by differences in the shape of the dorsomedial aspect of the internal structures and the intermediate levels at the caudal levels of the substantia nigra between PD and control subjects [7].

Finally, Bocti and colleagues applied a new MRI visual rating scale to assess white matter hyperintensities (WMH) within cholinergic pathways in subjects with Alzheimer’s disease (AD) and subcortical ischemic microvascular disease [11]. They found that the Cholinergic Pathways Hyperintensities Scale (CPHS) ratings were associated with cognitive impairment. This new MRI rating scale is reliable, shows stronger correlations with cognitive performance than a general WMH rating scale in AD with WMH, and could even be applied in advanced PD where the cholinergic deficit may contribute to cognitive decline.

Then, structural MRI also shows specific abnormalities in persons with atypical parkinsonism (APD), offering the possibility to distinguish the different forms of neurodegenerative parkinsonism [3]. In general, subjects with multiple system atrophy show a higher specificity of atrophy, signal changes in the putamen, and infra-tentorial structures on structural magnetic resonance imaging (sMRI). However, particularly in the early phases of the pathology, the sensitivity shows an unsatisfactory level [12]. Evidence suggestive of progressive supranuclear palsy (PSP), which is generally considered a tautopathy, has been reported, i.e., midbrain atrophy with enlargement of the third ventricle, reduced antero-posterior midbrain diameter and tegmental atrophy, signal increase in the midbrain and inferior olives, and atrophy of the frontal and temporal lobe [12]. In recent times, innovative benchmarks have been introduced to prospectively assess sensitivity and specificity of MRI measurements of the midbrain, pons, middle cerebellar peduncles (MCPs), and superior cerebellar peduncles (SCPs) and for differentiating PSP from PD and the Parkinson variant of multiple system atrophy (MSA-P), which are generally considered alpha-synucleinopathies [4]. SCP width and midbrain area in persons with PSP were statistically significant compared to subjects with Parkinson disease, subjects with MSA-P, and healthy subjects.

Little literature has deepened the role of structural MRI in persons with corticobasal degeneration (CBD) (another tautopathy). The data show cortical atrophy—particularly in frontoparietal areas—appearing asymmetric, putaminal hypointensity, and hyperintense signal modifications in the motor cortex or subcortical white matter on T2-weighted images [4].

### 2.2. Single Photon Emission Computed Tomography (SPECT) and Positron Emission Tomography (PET)

The diagnosis of parkinsonism is formulated on the basis of typical factors [13,14,15]. However, in older persons, diagnostic accuracy is questionable and the utilization of molecular imaging techniques, such as brain SPECT with [123I]FP-CIT, a dopamine transporter (DAT ligand) [16], or PET with 18F-fluorodopa (FDOPA) [17], can provide an increased accuracy of the diagnosis of idiopathic PD. It would also be possible to differentiate PD from other parkinsonian syndromes with uncommon symptoms or clinical course.

In PD, striatal binding of DAT is significantly lowered and is inversely proportional with the severity of the disease [18]. However, in literature, there is still debate about the clinical utility of DAT-SPECT, especially in terms of the cost–benefit ratio [19]. Several studies showed that a mild and asymmetrical reduction in [123I]FP-CIT uptake supports the diagnosis of degenerative PD or other atypical parkinsonism. However, the real clinical application of DAT-SPECT in the diagnostic path of patients with undefined parkinsonism is still questionable. Recently, the application of statistical parametric mapping (SPM) [18], allows the semi-quantitative assessment of the presynaptic dopaminergic uptake, showing a sensitivity and specificity for the diagnosis of presynaptic dopaminergic dysfunction that approaches 100% [18]. DAT-SPECT could add valuable knowledge in subjects with diagnostically ambiguous parkinsonism or tremor, particularly in the earliest phases of the pathology, although standardized algorithms should be applied for reinforcing the accuracy of this exam [20].

A recent longitudinal study, performed in PD patients, showed an independent association between a lower striatal binding at baseline and a higher risk for clinical outcomes and measures of PD severity, such as falling and postural instability, motor-related disability, cognitive impairment (CI), psychosis, and clinically relevant depressive symptoms. Subjects in the bottom quartile for striatal binding, compared to those in the top quartile, showed an odd ratio (95% confidence interval) of 3.3 (1.7–6.7) for CI and 12.9 (2.6–62.4) for psychosis [21]. Modifications in imaging after 22 months were independently associated with motor, cognitive, and behavioral outcomes.

Even if pronounced asymmetry in decreases of putamen DAT finding is more frequent for Parkinson disease than in other degenerative parkinsonism disorders, SPECT imaging using DAT ligands does not help in differentiating the typology of neurodegenerative parkinsonian disorders.

It seems that postsynaptic imaging can be helpful in the diagnostic process of PSP, thanks to a combination of [123I]FP-CIT with postsynaptic imaging using [123I]-iodobenzamide (IBZM)-SPECT. However, is not possible to exclude PSP in the case of a normal finding [22]. A reduction of [123I]-CIT uptake in the midbrain separate subjects with clinically fully developed MSA-P and PSP patients from PD. Nevertheless, subjects with subtle or ambiguous signs need to be investigated.

The frequent presence of vascular lesions and white matter basal ganglia ischemia in older persons may obstruct the distinction between vascular parkinsonism (VP) and PD [23]. In general, presynaptic dopaminergic circuitry is maintained in VP. However, a minor reduction in lateral SN has been reported, probably due to trans-neuronal degeneration together with moderate cell nerve loss in SN due to an important unilateral basal ganglia infarction. Persons with VP show a preservation or only a mild reduction in whole striatal [123I]-CIT binding and the putamen/caudate ratio. In the same way, VP subjects had intermediate binding between PD and control levels. In a sample of twenty persons with parkinsonism, with cerebrovascular disease, and without pre-existing PD, striatal [123I]FP-CIT binding showed a reduction in more than the 50% of the sample, in some cases with corresponding structural changes [24]. True nigrostriatal degeneration was proposed, but even with the use of in vivo striatal DAT markers, it may be difficult to recognize VP from PD [25].

Unconventional DAT-SPECT imaging supports nigro-striatal degeneration in subjects presenting parkinsonism. Normal DAT-SPECT supports a different diagnosis such as atypical parkinsonism, psychogenic- or drug-induced parkinsonism, vascular parkinsonism, dystonic tremor, essential tremor, or dopamine-responsive dystonia. An accurate and earlier detection can influence the therapeutics’ choices, and the cost-efficiency aspects of the healthcare systems.

We recently proposed a theoretical algorithm concerning the appropriateness of DAT-SPECT utilization by clinicians [20]. In detail, we emphasized the importance of the timely prescription of the DAT-SCAN for improving its sensitivity and specificity. The exam is also important for improving multi-domain clinical evaluation, especially motor and cognitive deficits, and choosing the most appropriate diagnostic tree in each single patient.

PET studies that evaluate brain metabolic differences described that a brain, which ages normally typically, shows a reduction in the cerebral glucose metabolism of the prefrontal cortex [26]. In PD patients, the regional glucose metabolism showed a reduction of 25% compared to control values for all brain regions. The major differences were found in the posterior brain areas (visual association cortex, primary visual cortex, and parietal cortex) and thalamus. It seems, therefore, that in PD patients without dementia, the cortical hypometabolism might primarily affect the posterior brain areas [27].

Recently, literature has shown that while non-demented patients with PD have a moderate cholinergic dysfunction, demented PD patients display higher cholinergic deficit in many cortical regions [28].

In PD, among the different non-dopaminergic neuronal systems affected, cholinergic cell loss was also found to be severe in the advanced stages of the disease, and recent studies have investigated the efficacy of anticholinergic drugs not only for treating Parkinson-Dementia, but also for reducing gait disorders [29].

Finally, in Parkinson’s disease dementia (PDD) not only could fluorodeoxyglucose (FDG)-PET and DATSCAN-SPECT be pathological, but also amyloid PET could show abnormal deposition of amyloid in several brain areas, producing mixed PD and AD diseases [30]. Atypical parkinsonism or Lewy body dementia (LBD) could benefit from different PET scans for a better diagnosis, such as FDG-PET and amyloid PET, given their heterogeneity of pathology [31,32].

### 2.3. Functional Magnetic Risonance Imaging (fMRI)

Recently, functional MRI (fMRI) has been widely applied in order to investigate the pathophysiology of motor and non-motor symptoms in PD. It seems that its early application can help in differential diagnosis and best treatment, and in predicting disease progression [33]. The fMRI has a greater safety profile than other imaging techniques, is easy to perform, and is widely common in hospital settings [33].

Nevertheless, this technique offers the possibility to measure the connectivity characteristics of different brain regions [33,34,35].

There is evidence that fMRI is useful in PD both with and without cognitive impairment.

Studies in patients with mild cognitive impairment (MCI) showed a reduced functional connectivity in accordance with the degree of cognitive impairment; in particular, patients with associated PD showed a reduced functional connectivity in the basal ganglia, ventral prefrontal, parietal, temporal, and occipital cortices [33,36].

Literature also shows that fMRI is a sensitive tool, which can detect brain network abnormalities in PD patients without obvious cognitive impairment, as well as some motor symptom features that imply higher risk for cognitive dysfunction in PD [33].

For instance, functional connectivity changes over time are correlated with the alteration of global cognitive function, as well as perception, praxis, and the spatial span subscores [33,37].

Recent longitudinal studies tried to define the trajectories of functional architecture changes according to PD stages and prognosis. With this purpose, Filippi et al. [38] applied the resting state functional MRI (RS-fMRI) in a sample of 146 PDs subjects in order to define their diseases’ subtype.

To be specific, moderate-to-severe PD subjects showed decreased mean nodal strength, local efficiency, and clustering coefficient, as well as a longer path length, compared to mild PD cases. The nodal properties changes involve sensorimotor, fronto-insular, temporal, and parietal regions. In particular, nodal strength can be considered the simplest estimator of the “wiring cost” of the network [38,39].

Finally, Filippi et al. identified four different patterns of progression: (1) a different trend of changes between subtypes (increase vs. decrease, increase or decrease vs. stable); (2) a similar trend of change (increase or decrease), with or without functional connectivity (FC) difference between the groups; (3) a different but stable FC in the two subtypes; and (4) a stable FC with no difference between groups [38].

## 3. Parkinson’s Disease Subtypes Identified by Tractography

Neuroimaging techniques are non-invasive and are capable of identifying the axonal pathways of human neural connections in vivo. In 1994, the development of diffusion tensor imaging (DTI) to study the organization of white matter in the living human brain represented a game changer [40]. For the first time, by this method, it was possible to measure and extract in vivo and non-invasively the organization and integrity of white matter fibers together with the quantification of the movement of water molecules inside the tissue. After years, tractography algorithms were proposed as a tool to mathematically reconstruct three dimensional trajectories of the major white matter pathways [41]. Different methods have been proposed to extract the fiber orientation directly, and the latest and most promising method is represented by using a specific diffusion model for white matter fibers. These approaches are usually described as spherical deconvolution methods, and they generally show a higher angular resolution (i.e., the ability to resolve crossing fibers at smaller angles) compared with methods based on diffusion orientation distribution functions (dODFs). This method has the advantage of requiring acquisition protocols that are close to clinical DTI protocols.

**Figure 1:**
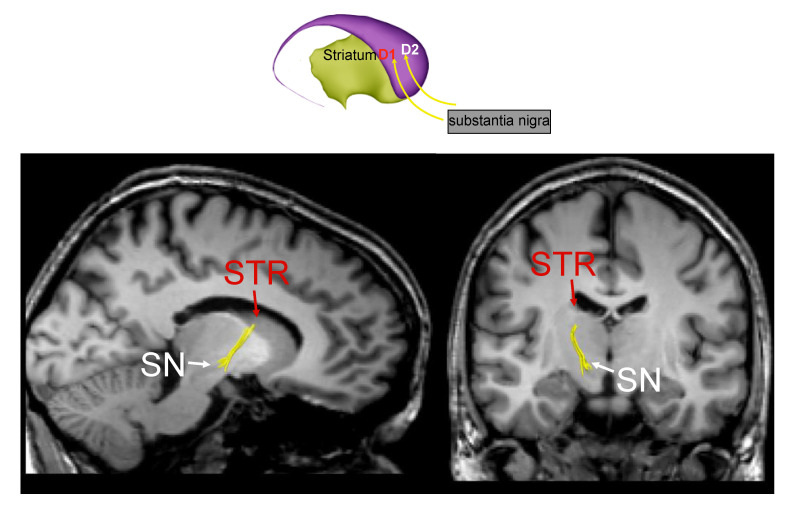
The fronto-striatal circuit (part a).

**Figure 2:**
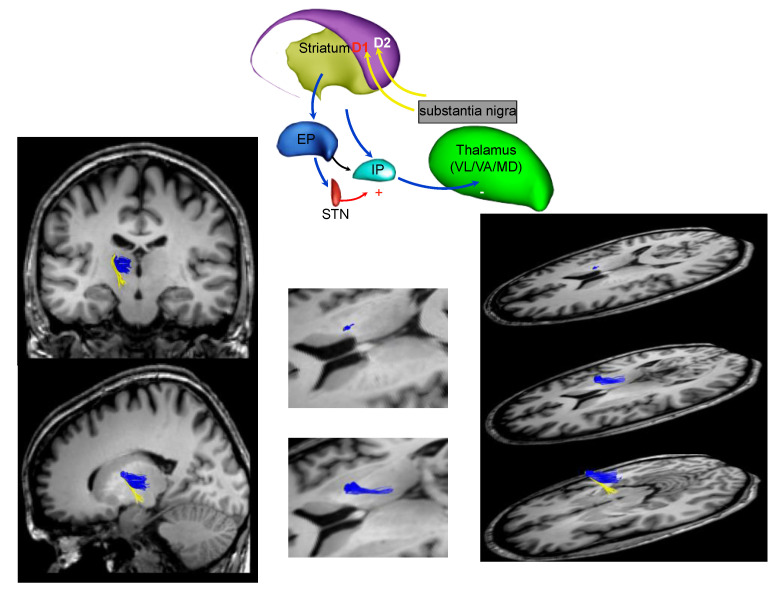
The fronto-striatal circuit (part b).

**Figure 3:**
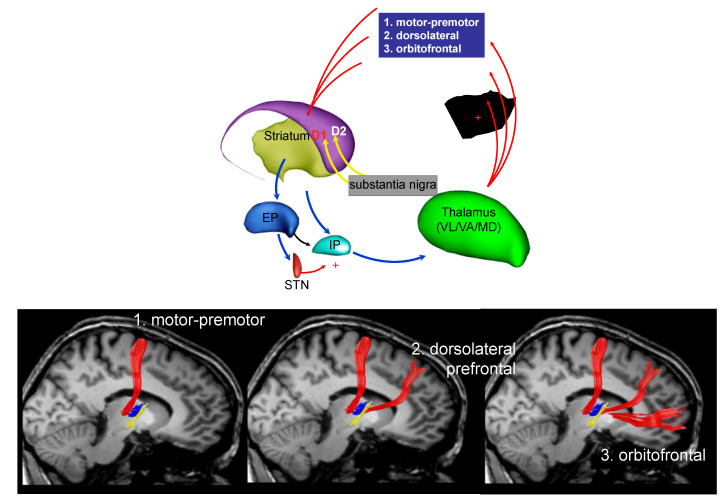
The fronto-striatal circuit (part c).

Using this technique, Catani and colleagues identified, for the first time in humans, pathways of the basal ganglia network using MRI tractography (Figure 1, Figure 2 and Figure 3) [42]. We show in Figure 1 the axonal connection between the SN and the striatum (STR). In Figure 2, we show the white matter fibers that connect the striatum (STR) to the thalamic nuclei. Alteration of these pathways could generate a loss of the reduced excitatory thalamic output to the cortex, with development of the extrapiramidal signs of PD.

Figure 3 shows connection between the thalamic nuclei and the cortex: (1) from the thalamic nuclei to the motor and premotor cortex, (2) from the thalamic nuclei to the dorsolateral prefrontal cortex, and (3) from the thalamic nuclei to the orbitofrontal cortex. An alteration of these connections could explain motor, cognitive, and behavior symptoms in PD [43].

Subtypes of PD have been identified, first clinically and then statistically. They show superimposable characteristics at presentation of the diseases and an often comparable response to the levodopa treatment [44,45]. Even the progression of the disease is similar for each PD subtype, making more understandable the importance of characterized PD phenotypes for improving the history of this disease.

The cluster analysis has been used for reproducing the clinical impression of the existence of specific PD subtypes, and a recent study found that at least four subtypes of PD exist, with patients that share homogenous features: (1) rapid disease progression and old age at onset; (2) slow disease progression and young age at onset; (3) tremor dominant; and (4) dominance of bradykinesia/rigidity or postural instability and gait disorder (PIGD) [46]. Discordant results for this cluster profile were found with respect to the association with CI (unaffected, mildly impaired, and impaired) and depressive symptoms (mild and severe).

The tractography could be considered as a precious technique to reach the characterization of PD subtypes, given that, as previously shown, it permits the depiction dopaminergic pathways and allows us to unify clinical features, behavior, and functional neuroanatomy of PD.

**Figure 4:**
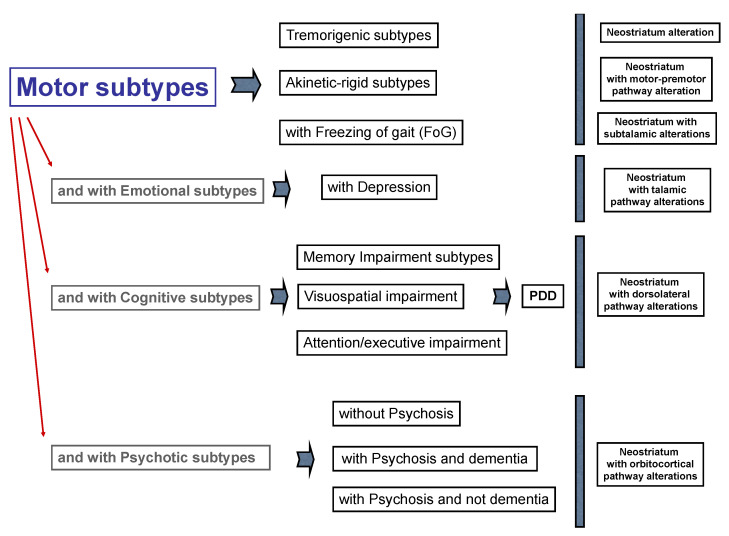
Types of Parkinson’s disease (PD) integrating motor and non-motor symptoms and fronto-striatal circuit alteration.

In Figure 4 we propose a schematic representation of possible isolated phenotypes of PD that could be characterized by DAT-SPECT and MRI tractography in terms of extension of neuroanatomical brain compromising. We hypothesize, for example, that the “PD subtype with freezing of gait” shows a striatal alteration, assessed by DAT-SPECT, plus a destroyed connection with the subthalamic nucleus. When patients show motor complaints plus depressive symptoms, a neostriatum with thalamic abnormal connection should be hypothesized. Then, when patients are affected by motor complaints and cognitive impairment, an alteration between the neostriatum and dorsolateral pathway could be identified. Finally, in patients with motor complaints plus psychotic features, an alteration between the neostriatum and orbitocortical pathway could be observed.

It is noteworthy that if we identify the exact functional neuroanatomy of Parkinson’s disease subtypes in vivo, we could precisely assess and predict the efficacy and side effects of dopaminergic drugs (especially dopamine receptor agonists), such as gambling, hyper-sexuality, and compulsive shopping [47].

Recently, clinicians have emphasized that non-motor symptoms in PD should be treated with specific caveats [48]. For example, depression in PD may benefit from the optimization of dopaminergic therapy, from the use of antidepressants acting on both the serotoninergic and noradrenergic pathways. Psychosis in PD may improve only with the use of clozapine and quetiapine. First at all, treatment of impulse control disorders should be based on the removal of dopamine agonists. Hallucinations may benefit from reducing the daily dosage of levodopa, while there is no evidence on the treatment of neuropsychiatric disorders in multiple system atrophy, progressive supranuclear palsy, or corticobasal degeneration. Finally, acetylcholinesterase inhibitors may be used to treat mild cognitive impairment in PD.

Levodopa use has been recently confirmed as the gold-standard of the treatment. The age of the subjects [49] and the subtypes of the PD should be considered for identifying the daily dosage of levodopa. In fact, long-term, double-blind studies have demonstrated that dosage is a relevant risk factor for the onset of motor fluctuations and dyskinesia, and suggest the lowest possible doses of L-dopa [50].

Although drug therapy is of fundamental importance for the treatment of PD, the adherence to the therapeutic plan is often sub-optimal [51].

The side effects of some medications (i.e., L-dopa), the individuals’ beliefs about their disease, and their therapeutic plan may affect the adherence, especially in the initiation phase [52].

It has been reported that a relationship of trust with the reference health care professionals can improve the patients’ compliance. In this regard, nurses may play a fundamental role [52].

As PD is often associated with dysphagia, the consequent swallowing difficulties may affect the consumption of prescribed medications; however, patients may be reluctant to admit these difficulties [53].

In this perspective, community nurses could actively screen dysphagia in these patients and, if confirmed, provide a first line education to the person and the caregiver, if present [53].

Nevertheless, recent guidelines suggest the adoption of the Parkinson’s disease nurse specialist (PDNS) [54]. This professional can improve patients’ general well-being, physical function, and the overall health status, as well as reducing anxiety and depression [55].

The PDNS can also lead and monitor comprehensive interventions in a community setting, with non-pharmacological interventions such as physical exercise [56], which may be very useful in reducing the risk of fall-related fractures and the subsequent hospitalization [57].

Important is also the utilization of specific drugs when a patient with PD is admitted into hospital and develops delirium. In this case, only specific drugs could be used for avoiding a worsening of parkinsonian symptoms [58], but often, these caveats are not followed.

## 4. Conclusions

In conclusion, the subtyping of PD patients is not a mere clinical classification but reflects different pathophysiological mechanisms with different risk factors [59,60], and an integration between different neuroimaging techniques could improve the characterization and treatment of this “salamander” disease.

## Data Availability

Data are available asking to Marco Catani of the Natbrainlab at the King’s College of London.
